# Using DNA Metabarcoding and Citizen Science to Assess the Diet of Owned Domestic Cats

**DOI:** 10.1002/ece3.74404

**Published:** 2026-09-21

**Authors:** Hee Jin Chung, Stevi L. Vanderzwan, Benjamin N. Sacks

**Affiliations:** ^1^ Department of Environmental Science and Policy University of California Davis California USA; ^2^ Veterinary Genetics Lab, Mammalian Ecology and Conservation Unit University of California Davis California USA

## Abstract

Various methods have been attempted to quantify hunting and consumption of wild prey by domestic cats, but molecular analysis of their scat using DNA metabarcoding has rarely been attempted. In this study, we relied on owner‐collected scats (*n* = 32) from litterboxes and backyards to test whether this methodology could be used to detect consumption of wild prey by owned, indoor–outdoor domestic cats, and whether pooling scat samples could enhance efficiency or would result in a loss of resolution. We monitored eight cats for an average of 12.1 days, collecting an average of four scats from each. Metabarcoding revealed only one instance of wild prey consumption, a California vole ( *Microtus californicus* ), despite the owner not witnessing any prey returns during the 24‐h period prior to scat sampling. Conversely, in three instances where the owner reported returns of intact prey by their cats, prey DNA was not recovered, presumably because no prey parts were consumed. Thus, consumption and owner‐reported capture of prey may be poorly correlated. Pooling scats did not influence detection of ubiquitous, abundant taxa such as chicken contained in their owner‐provided food, but consumption of a vole by one cat was only detectable in the scat tested alone. Pooling scats for analysis may sacrifice sensitivity to detection of wild prey. Our study suggests that DNA metabarcoding may be a viable method to detect consumption of wild prey by owned domestic cats; however, a larger sample size is needed to investigate the biodiversity of taxa in the diet, as the frequency of prey consumption by cats may be lower than that of prey capture.

## Abstract 2

This small‐scale study was conducted to test the viability of DNA metabarcoding as a method to detect prey consumption by owned domestic cats. We found that owner reports and scat analysis showed decoupled evidence of hunting behavior, and that pooling scat samples prior to DNA extraction reduced the resolution of wild vertebrate prey detected, but not of ubiquitous livestock species such as chicken and pig.

## 1 Introduction

Non‐native predators have the potential to impact naïve local wildlife (Carthey and Banks 2014; Jakubavičiūtė and Candolin 2021; Sih et al. 2010), especially when in increased densities or abundance due to human activity (Early et al. 2016; Pyšek et al. 2010). One such predator of concern is the domestic cat, for which hunting tendencies have been thought to cause both consumptive and nonconsumptive effects on vulnerable prey species, such as changing prey physiology and behavior (Beckerman et al. 2007; Bedoya‐Pérez et al. 2021; Bonnington et al. 2013), population decline (Loss et al. 2013; Loss and Marra 2017; van Heezik et al. 2010), and even species extinction (Crooks and Soulé 1999; Doherty et al. 2016; Medina et al. 2011). Unlike most other predators, however, domestic cats also play a significant role in human society as companion animals (Crowley et al. 2020a, 2020b; Jaroš 2016). Given their relationship to humans, cats may transmit zoonotic disease (Roseveare et al. 2009; Szentivanyi et al. 2024), and unique challenges exist when monitoring their impact and planning sustainable conservation solutions (Cecchetti, Crowley, and McDonald 2021; Crowley et al. 2020a, 2020b; MacDonald et al. 2015).

One major goal in mitigating the impact of domestic cats (“cats” hereafter) on wildlife is monitoring and quantifying their hunting behavior. Various methods traditionally used in wildlife monitoring have been attempted in domestic cats (Lockwood and Huck 2025), such as camera traps (Abrahams et al. 2025; Herrera et al. 2022), cat‐borne cameras (Hernandez et al. 2018; Loyd et al. 2013), manual assessment of stomach contents or scats (Krauze‐Gryz et al. 2012; Piontek et al. 2021), and stable isotope analyses (Cecchetti, Crowley, Goodwin, Cole, et al. 2021). In addition to the typical challenges associated with these methods (e.g., selection bias, limited battery power, and lack of resolution), some of these methods may also face pushback from the public when implemented for cats, particularly when it is viewed that cat welfare may be compromised (e.g., Crowley et al. 2020a, 2020b; Linklater et al. 2019). Cat owner‐reported observations of hunting may mitigate some of these concerns (e.g., Cecchetti, Crowley, Goodwin, and McDonald 2021; Chung et al. 2024; Kauhala et al. 2015), but the reliability of such data may be questionable (Pongrácz et al. 2024). It is therefore crucial to test alternative, noninvasive methods of monitoring cat impact on wildlife that involve active participation from caretakers of domestic cats.

One method that has been underutilized in research is dietary analysis of cat scats. Scats provide a noninvasive method of monitoring diet and are regularly produced by animals, ensuring sample availability. While visual or morphological analyses of scats may allow for identification of some prey, morphological analysis often lacks the resolution needed to distinguish to the species level (Galão et al. 2025). DNA Metabarcoding is a widely used method of monitoring DNA composition of samples such as scats and can provide high resolution data up to the species level (De Barba et al. 2014; Pompanon et al. 2012; Shehzad et al. 2012; Swankhuisen et al. 2026; Thuo et al. 2019; Van Der Heyde et al. 2021). Diet metabarcoding may thus provide a reliable, high‐resolution, noninvasive alternative to existing methods of quantifying cat hunting.

One potential barrier to molecular methods of diet analysis is the cost associated with DNA extraction and sequencing. Pooling independently‐collected scat samples within an individual animal prior to DNA extraction may reduce the total number of extractions to be performed. While studies have assessed some optimization techniques for DNA metabarcoding (McInnes et al. 2017), no study to our knowledge has systematically compared the overall resolution of data obtained when extracting and amplifying DNA separately from individual samples versus from pooled combinations of those samples. We tested whether pooling independent samples from an individual cat prior to extraction would maintain similar representation of the animals' diet. While DNA metabarcoding has been used to assess the diet of feral cats (Galão et al. 2025; Plimpton et al. 2021), no study to our knowledge has tested this methodology on owned cats. Owned cats are an important population to be studied, given that those allowed outside continue to exhibit hunting behavior (Bradshaw and Cook 1996; Driscoll et al. 2009). While most owned cats are sterilized and regularly fed, neither sterilization nor regular feeding has been shown to reduce hunting behavior of cats on an individual level (Cecchetti, Crowley, and McDonald 2021; Guttilla and Stapp 2010). Cats are known to exhibit hunting behavior even when satiated (Loyd et al. 2013), potentially for enrichment, practice, or caching when hunting opportunities are available (Adamec 1976; Cecchetti, Crowley, and McDonald 2021). Targeting owned cats allows collection of detailed individual‐level data, and can utilize citizen science to obtain scat samples, directly reducing the substantial effort and costs associated with sample collection. Due to their hunting beyond nutritional need, cats do not always consume the prey they capture (Adamec 1976; Cecchetti, Crowley, and McDonald 2021); owner reports can therefore provide additional methods of monitoring cat hunting behavior and allow for comparisons between what is captured and consumed versus unconsumed. Given that they are fed by their caretakers, owned cats can also allow a direct comparison between what the cat consumes (e.g., their provisioned food) and what is found in their scats. We therefore sought to investigate whether owned cats that regularly produce scats in the home environment, either in a litter box or an outdoor yard, could provide a useful study system to assess the impact of this predator on local wildlife.

Here, we use DNA metabarcoding to assess the diet of owned domestic cats in the Davis and Sacramento regions of California, USA. We designed this study to pilot test this method's viability as a tool to capture consumption of wild prey by owned domestic cats despite their reliance on humans and consumption of human‐provisioned cat food. We recruited owners of indoor–outdoor cats to record their cat's observed hunting behavior and collect samples of their cat's scats and provisioned food. We had three main aims for this study: (1) To assess if DNA metabarcoding could be used to detect wild prey DNA in the scat of owned, indoor–outdoor domestic cats; (2) to collect cat food samples that were being fed to our study cats and compare DNA found in their foods vs. in their scats; and (3) to test a modified sampling technique where extractions from individual fecal samples are compared to extractions from pooled samples. We predicted that scats of owned indoor–outdoor cats would show the presence of wild prey species, especially if the cat was seen hunting wild prey by their owner. Alternatively, if cats that hunt prey for food are more likely to consume prey out of the owner's vision than cats that capture prey for play, it is possible that the two types of hunting are decoupled.

## 2 Methods

### 2.1 Ethical Note

Cat owners who participated in another study (Chung et al. 2024) were invited to provide scat samples of their cats at the end of the study. A consent form was provided at the beginning of the questionnaire for the previous study, and consenting participants were asked to provide an email address for coordination of pickup of the scat samples.

This study was reviewed and determined exempt (nonhuman subjects research) by the Institutional Review Board of the University of California, Davis (#1905569). This study did not require approval from the Institutional Animal Care and Use Committee of the University of California, Davis, as it did not involve direct interaction with, handling, or manipulation of any animals.

### 2.2 Sample Collection

In total, 65 owners were invited to participate in this study, 49 of which responded to the initial contact, and 17 of which received the sample collection kit. We successfully received scat samples from 8 of the participating owners. Of the surveyed cats, 3 were male and 5 were female, and all were neutered or spayed (Appendix: Table S3). For anonymity, the cat subjects are labeled as Cat A–H. For sample collection, we provided a small collection kit to each participating owner with the following: (1) five 50‐mL falcon tubes including approximately 25 mL of 95% ethanol, (2) five pairs of nitrile gloves to aid in sample collection, and (3) printed instructions including a study log to record the date, time, and place the sample(s) were collected, and whether their cat was seen with a caught wild animal within the past 24 h of each collection. The collection kit was dropped off at the location the participant provided, and later picked up upon the owner's completion of sample collection. We directed each owner to provide five scat samples from their cat and to store them in the falcon tubes provided at room temperature. Along with scat samples, we also requested a sample of their cat's food that was currently being provisioned in the home. In total, we collected 38 scats from eight different cats in Davis and Sacramento, CA, USA, from 8 November to 21 December 2023.

In addition to the scat collection kit, participating cat owners were also provided a physical log to record the following metadata associated with each scat sample: date collected, time collected, approximate time the scat was produced, location of the scat, number of hours the cat spent outdoors that day, and details on any observed instances of hunting within 24 h of the scat collection (e.g., observed captures of prey and/or carrying of prey items). These logs were collected with the scat and food samples for assessment.

### 2.3 Laboratory Procedure

We conducted all laboratory analyses at the Mammalian Ecology and Conservation Unit of the Veterinary Genetics Laboratory at University of California, Davis from December 2023 to July 2024. We extracted DNA from all samples using the EurX Stool DNA Purification Kit (EurX, Gdansk, Poland) following the manufacturer's instructions, except that DNA was eluted in a 55 μL volume to increase DNA concentration.

During the scat teasing process, we created pooled samples of each cat's scats by homogenizing small subsamples of each of the 3–5 individual scats into a combined, pooled scat sample. We avoided subsampling the external layer of each scat and the hair, as these were most likely to contain host DNA rather than their diet. A sample of the pooled scat was then collected twice and treated as two additional samples during extraction.

We prepared two plates, one for each primer set (12S and 16S), and included three concentrations (1×, 10×, 100×) of the positive controls, along with five PCR‐negative controls and seven extraction blanks, one from each extraction event. For 12S, we included a mixture of positive controls containing equal concentrations (0.002 ng/μL) of DNA of each of the following vertebrate species, which represented a variety of taxa both local and not local to the study area: Desert cottontail (*Silvilagus audubonii*), Laysan albatross ( *Phoebastria immutabilis* ), Northern elephant seal ( *Mirounga angustirostris* ), yellow‐backed spiny lizard ( *Sceloporus uniformis* ), western whiptail lizard (*Aspidoscelis tigris*), western harvest mouse ( *Reithrodontomys megalotis* ), western fence lizard ( *Sceloporus occidentalis* ), alligator lizard ( *Elgaria multicarinata* ), and blunt nosed leopard lizard ( *Gambelia sila* ). Several species of lizards were included, as we predicted reduced amplification of herptile DNA using 12SV5. For 16S, we included a mixture of positive controls containing equal concentrations (0.002 ng/μL) of DNA of each of the following invertebrates: moth (*Lepidoptera* spp.), stiletto fly (*Thereva* spp.), pholcid spider (*Pholcidae* spp.), and whiteleg shrimp (*Penaeus vannemei*). We included several PCR‐negative controls and extraction blanks to assess background contamination. For the remaining 23 wells, we selected an arbitrary set of samples to replicate as PCR replicates to further ensure repeatability of our results (Appendix: Tables S1 and S2).

We prepared libraries in three steps, following protocols outlined by Caspi et al. (2025). First, we performed an amplicon PCR targeting the V5 loop of the mitochondrial 12S gene and included sequence overhangs to 5′ ends to allow annealing to Illumina index sequences in the index PCR. To reduce amplification of host DNA, we designed a blocking oligonucleotide specific to domestic cats using previously described methods (Shehzad et al. 2012; Vestheim and Jarman 2008). The blocker sequence was CTATGCTTAGCCCTAAACTTAGATAGTTACCCTAAACAAAACTATC/3SpC3/, where 3SpC3 is a 3′ spacer of three carbons that prevents further synthesis along the template strand, and therefore prevents amplification. For 16S, we amplified the mitochondrial 16S rRNA gene using universal primers, IN16STK‐1F: TGAACTCAGATCATGTAA and IN16STK‐1R: TTAGGGATAACAGCGTAA (Kartzinel et al. 2015), except that we included 5′ tags on each primer enabling annealing to Illumina adapters during a second PCR. Due to limited resources, we prioritized maximizing inclusion of biological replicates (different scats of the same individual) rather than technical replicates (PCR replicates), given that biological replicates have been shown to yield more variation in diet compared to technical replicates (Mata et al. 2019). We performed the amplicon PCR with a thermal profile of 95° for 15 min, then 40 cycles of 30 s at 94° and 90 s at 55°, and then held at 15°.

Second, we purified the PCR product using an Exo‐SAP‐IT reaction (Caspi et al. 2025). The reaction consisted of 3 μL of amplicon PCR product, 2.78 μL of 1× TE (10 mM Tris–HCl + 1 mM EDTA), 0.075 μL of 20 U/μL Exonuclease I, and 0.15 μL of 1 U/μL shrimp alkaline phosphatase (New England Biolabs, MA, USA). The reaction was then incubated for 1 cycle of 37° for 30 min and 1 cycle of 80° for 15 min. Finally, we performed an index PCR to anneal Illumina sequencing‐compatible adapters and assign unique index pairs to each sample. The index pairs included one 8‐bp i5 index sequence assigned to the plate (96 samples including controls), and one 6‐bp i7 index sequence assigned to each sample within the plate. The index PCR was run for 1 cycle of 98° for 30 s, 8 cycles at 98° for 10 s and 65° for 75 s, and 1 cycle of 65° for 5 min, then held at 4°.

### 2.4 Bioinformatic Processing

Upon recovery of sequences, we trimmed and filtered out low‐quality sequences using cutadapt (Martin 2011). We identified Amplicon Sequence Variants (ASVs) using DADA2 (Callahan et al. 2016), and used the blastn feature of BLAST+, as well as the assignTaxonomy feature of DADA2 to assign ASVs to the custom reference libraries. We then used the basic local alignment search tool (BLAST) in NCBI to confirm assignments, or to assign to species not included in the reference libraries (Zhang et al. 2000). We assigned ASVs at the species or genus level whenever possible, though most invertebrate taxa could not be identified beyond the Order. The majority of our ASVs showed 100% query coverage and matched at 98%–100% identity. In the case where the ASV matched with more than one species, or were matched with species not found in northern California, we used iNaturalist (www.inaturalist.org) to identify the species found in the local areas, with the exception of species likely to be found in commercially available pet foods.

Upon assigning taxonomy, we removed ASVs that comprised < 0.001% of the total sequence reads in a lane, and ASVs with low percent identity (< 97% for 12S, < 94% for 16S) and/or query coverage (< 98%), which removed 24 ASVs in total (15.5%). We then concatenated by taxa to combine ASVs assigned to the same species. We used positive and negative controls to determine filtering thresholds (minimum numbers of reads), and discarded samples failing to exceed them (848 reads for 12S and 16,582 reads for 16S). Upon filtering for successful amplifications, we removed non‐diet items, including host (cat) DNA, human, and domestic dog. After removing non‐diet items, we then removed diet items representing < 0.5% of reads within a scat. Diet items were grouped into anthropogenic (items expected in provisioned pet food), wild prey, and arthropods (16S).

### 2.5 Domestic Cat Diet Analysis

We performed all analysis and visualization in the statistical software program R (v4.4.3; R Core Team, 2022). We turned raw sequence reads into frequency of occurrence, calculated by the number of occurrences of an item divided by the total number of samples. We compared the presence/absence of each diet item across individual cats, and for those whose provisioned pet food samples were available, compared the presence/absence of the diet items found in the provisioned food against the scat samples that were collected. We then compared the presence/absence of diet items found in individual scat samples against the pooled samples, to assess the consistency of recovered diet items between pooled and un‐pooled samples. Although we designed a blocking oligonucleotide to reduce amplification of cat DNA in our samples, the majority of DNA found in each scat sample still remained cat DNA, which is not unusual (Shehzad et al. 2012).

## 3 Results

Scats were collected for the duration of 12 days per cat on average (range = 1–25 days, SD = 9.5 days, Appendix: Table S4). All scat samples were relatively fresh, collected within an average of 5.5 h post defecation (range = 1–960 min, SD = 289.02 min, Appendix: Table S3). The majority (94.7%) were collected from a litterbox, with only two samples (both from Cat C) collected from the backyard.

We retained a total of 32 individual samples from eight cats after filtering for amplification success, along with two replicate pooled samples for each cat. One individual scat (from Cat C) was identified as an opossum (*Didelphis virginianis*) scat misidentified by the owner as a cat scat and was subsequently removed from the dataset, along with the two pooled samples it was associated with (Cat C). While eight other scats contained opossum DNA, we suspected possible contamination from the opposum scat and excluded opossum from further analysis.

### 3.1 Note on Invertebrate DNA

Invertebrate diet items recovered with 16S primers suffered from significant contamination (> 8000 raw sequence reads) in negative controls and low amplification across samples. Overall, we retained three cat food samples, six individual scat samples from two cats, and three pooled samples after filtering for amplification success. All scat samples that were retained contained Orthoptera (Appendix: Table S5). Diptera (flies), Hemiptera (true bugs), Salticidae (jumping spider), and Noctuidae (noctuid moths) were also found in cat scats. From cat food samples, we identified Orthoptera (grasshoppers and crickets), Coleoptera (beetles), Hemiptera, Noctuidae, Hymenoptera (wasps, bees, and ants), Cephalopoda (cephalopods), and Diptera. Given the low resolution of data, we excluded 16S data from further examination.

### 3.2 Presence of Wild Prey

Despite our study cats being fed animal‐based pet foods, about a third (35.5%) of our scat samples contained no vertebrate DNA except cat DNA. Throughout the study period, there were three instances where the cat was observed by the owner carrying a wild prey item within 24 h prior to each scat collection: a house mouse ( *Mus musculus* ) caught in the home, a small rodent of unknown species brought to the door, and a lizard of unknown species caught in front of the home (Appendix: Table S6). In all three instances, the owner reported that the prey remained alive and intact, and the scats that were produced by the cat within 24 h of the hunting event showed no presence of wild prey. Of all 32 scat samples, only one scat (from Cat H) showed presence of wild vertebrate prey DNA, which was California vole ( *Microtus californicus* ). The cat that produced this scat was not seen by its owner with any wild prey within 24 h prior to the collection of this scat. Vole was only detected in one scat sample of this cat and was not seen in any subsequent scat samples produced by this cat (Appendix: Table S3).

### 3.3 Provisioned Foods vs. Scats

We were able to collect samples of the foods that were being provisioned to four of our study cats (Cats A, B, D, E). Overall, six taxa of anthropogenic origin were identified in these four cats' diets: chicken, pig, turkey ( *Meleagris gallopavo* ), and fish in the drum and croaker family (Sciaenidae), hake family (Merluccius), and herring family (Clupeidae) (Table 1). Chicken and pig (or pork) were found in all four food samples. Chicken had the highest rate of recovery after digestion, with 14 out of the 15 expected scats showing its presence. Fish (specifically, Sciaenidae) was the only taxon of anthropogenic origin in the scat that was not also found in the food sample; we presume that this may be due to a supplemental food source containing fish, such as a treat. Several scat samples showed no animal DNA other than cat DNA (Table 1).

**TABLE 1:** Comparisons between vertebrate (12S) diet items recovered from scat samples (*n* = 15) produced by a subset of cats (*n* = 4) vs. diet items found in the pet food provisioned to those cats in Davis and Sacramento, CA, USA.

	Chicken	Pig	Turkey	Fish (Clupeidae)	Fish (Sciaenidae^a^)	Fish (Merluccius)
Cat A—Food (HP^b^, dry)	✓	✓		✓		
Cat A—Scat Sample 1						
Cat B—Food (kidney care^c^, wet)	✓	✓				
Cat B—Scat Sample 1						
Cat B—Scat Sample 2						
Cat B—Scat Sample 3						
Cat B—Scat Sample 4						
Cat B—Scat Sample 5	✓					
Cat D—Food (12+^d^, wet)	✓	✓				
Cat D—Scat Sample 1	✓					
Cat D—Scat Sample 2	✓					
Cat D—Scat Sample 3	✓					
Cat D—Scat Sample 4	✓					
Cat D—Scat Sample 5	✓	✓				
Cat E—Food (chicken and turkey, dry)	✓	✓	✓			✓
Cat E—Scat Sample 1	✓	✓			✓	
Cat E—Scat Sample 2	✓	✓				
Cat E—Scat Sample 3	✓					
Cat E—Scat Sample 4	✓	✓	✓			
Total	14/15	4/15	1/5	0/1	N/A	0/5

*Note:* Highlighted rows indicate the pet food, and ✓ indicates presence of the taxon in the food or scat sample. “Total” indicates the following: (total number of observed presences of the taxon in the scat)/(total number of expected presences of the taxon in the scat based on its presence in the food). Samples listed above that are blank indicate that no vertebrate taxa were found in the sample except cat DNA. Some data are excluded (cats C, F, G, and H) due to lack of availability of their provisioned food samples.

^a^ Sciaenidae was found in one of the scat samples of Cat E, but was not present in the provisioned food sample.

^b^ HP indicates prescription hydrolyzed protein.

^c^ Kidney care indicates prescription kidney care food.

^d^ 12+ indicates food formulated for senior cats 12 years or older.

Vertebrate diet items found in food samples A, B, D, and E were recovered in scat samples of the corresponding cats to varying degrees (Table 2). Only two samples showed presence of identical taxa between scats and the food (Cat D—Scat Sample 5, and Cat E—Scat Sample 4, Table 1). Given that the food samples were collected from the owners, the food types were outside of our control and naturally differed in their type (wet/dry), formulation, and composition (Table 2). Food A was a prescription hydrolyzed protein cat dry food, designed for cats intolerant of or allergic to common proteins such as chicken and beef; food B was a prescription kidney care wet food; food D was wet food formulated for senior cats 12 years and older; and food E was a chicken and turkey based dry food. More details on the exact formulation and ingredients for each food can be found in the Appendix (Table S7).

**TABLE 2:** Proportions of vertebrate diet items in provisioned foods A, B, D, and E that were recovered in the scats (*n* = 15) produced by corresponding cats (*n* = 4) living in Davis and Sacramento, CA, USA.

	Proportion of diet items in food that were recovered in scat
Food A: Prescription hydrolyzed protein dry food	0/3
Food B: Kidney care, wet food	1/2
Food D: Senior dry food	2/2
Food E: Chicken and turkey, dry food	3/4
Total	Range = 0–1, mean = 0.563, SD = 0.370

### 3.4 Pooling Scats

We found varying levels of similarity between individually‐extracted scat samples and pooled‐extracted samples (Table 3). Overall, individually extracted scat samples yielded more diet item detections than did pooled‐extracted scat samples, with the exception of one cat (Cat D); Cat D showed more diet items in pooled samples than individually extracted samples (Sciaenidae, turkey, and opossum). While it is possible this occurred due to pooled samples containing parts of the scat not represented by individually‐extracted samples, we suspect that contamination may have occurred from samples from Cat E, due to their proximity to each other on the PCR plate. Seven individually extracted samples and three pooled‐extracted samples showed no animal DNA except cat DNA (Appendix: Table S4).

**TABLE 3:** Comparisons between diet items recovered in individually extracted scat samples of each cat (*n* = 28) vs. diet items recovered in pooled‐extracted samples (*n* = 13) within each cat of our sample in Davis and Sacramento, CA, USA.

Individually extracted vs. pooled									
	Chicken	Pig	Turkey	Fish (Merluccius)	Fish (Sciaenidae)	Fish (Salmonidae)	Mackerel	California vole	Opossum
Cat A: Individual (*n* = 1)									
Cat A: Pooled (*n* = 2)									
Cat B: Individual (*n* = 5)	✓								✓
Cat B: Pooled (*n* = 2)	✓								✓
Cat D: Individual (*n* = 5)	✓	✓							
Cat D: Pooled (*n* = 2)	✓		✓		✓				✓
Cat E: Individual (*n* = 4)	✓	✓	✓		✓				✓
Cat E: Pooled (*n* = 2)	✓	✓	✓		✓				✓
Cat F: Individual (*n* = 5)	✓	✓		✓		✓			✓
Cat F: Pooled (*n* = 1)	✓					✓			
Cat G: Individual (*n* = 4)	✓	✓	✓		✓		✓		✓
Cat G: Pooled (*n* = 2)	✓	✓							✓
Cat H: Individual (*n* = 4)	✓		✓					✓	✓
Cat H: Pooled (*n* = 2)	✓								
Total ratio	6/6	2/4	1/3	—	1/2	1/1	0/1	—	3/5

*Note:* “Individual” indicates all diet items recovered from individual scat samples of each cat. “Pooled” indicates all diet items recovered from extractions performed on homogenized pooled samples from the corresponding cat. ✓ indicates the presence of the taxon in the sample. A greater match between Individual and Pooled data suggests that pooling samples prior to DNA extraction did not reduce resolution of the data. Total ratio = (number of times the diet item was observed in individually‐extracted samples)/(number of times the diet item was observed in pooled‐extracted samples). Any deviation from *n* = 5 for “Individual” and *n* = 2 for “Pooled” samples is due to failed amplification of DNA. Cat C was excluded from this table due to one of its samples being identified as opossum scat that contaminated all of its pooled samples.

Chicken was always recovered from pooled samples when it was present in individually‐extracted samples (Table 3). In contrast, vole, a wild prey species, was found in an individually extracted scat sample (Cat H) but not recovered when the sample was pooled with other scats. Raw sequence reads can be found in the Appendix (Table S8).

Overall, pooled extractions had varying degrees of success in amplifying diet items that were recovered in individually extracted samples (Table 4). Samples from two cats (cats B, E) showed a perfect match between diet items recovered from individually‐extracted versus pooled‐extracted samples. Raw sequence reads can be found in the Appendix (Table S9).

**TABLE 4:** Proportions of diet items found in individually extracted samples that were recovered in pooled‐extracted samples of scats produced by seven cats in Davis and Sacramento, CA, USA.

	Proportion of diet items in individually extracted samples that were recovered in pooled samples
Cat A	N/A
Cat B	2/2 = 1.0
Cat D	1/2 = 0.50
Cat E	5/5 = 1.0
Cat F	2/5 = 0.40
Cat G	3/6 = 0.50
Cat H	1/4 = 0.25
Total	Range (0.10–1.0), mean = 0.608, SD = 0.316

*Note:* Diet items that were found in pooled samples but not found in individually‐extracted samples were excluded to assess only the effect of pooling methods on the resolution of data obtained.

## 4 Discussion

### 4.1 Presence of Wild Prey in Diet

In this sample of cats and their owners, contrary to our predictions, we found that owner reports of catching and handling wild prey by cats did not coincide with DNA found in their scats. We detected no wild prey DNA in the scats of cats that were seen with a prey item within 24 h prior to scat collection. This finding is likely attributable to the prey having been unconsumed, as owner‐reported observations showed prey intact and alive. Although it is possible that trace amounts of prey DNA could be ingested without the need to consume prey (e.g., hairs that become dislodged and swallowed when mouthing prey), our findings suggest that consumption of significant prey parts by cats may be necessary for detection of prey DNA in their scats. Interestingly, of the 32 scats collected, only one scat showed any presence of wild prey in its diet, and this cat was not seen with any wild prey by its owner within 24 h prior to the collection of this scat. Cats often hunt without consuming prey (Loyd et al. 2013; McGregor et al. 2015), and tend to consume certain types of prey and return others to the home (Lockwood and Huck 2025); our data show preliminary support for using DNA metabarcoding to capture instances of hunting where the cat has consumed the prey upon capture instead of returning home with intact prey. We suggest that owner reports and molecular analysis of scats should be used in combination by future studies in order to capture multiple types of hunting done by owned, indoor–outdoor cats.

Aside from opossum, which we suspect to be contamination from the one scat processed with them and later identified as an opossum scat, the only wild prey detected was vole. This was not surprising given that voles are commonly found in this region, specifically *Microtus californicus* (http://inaturalist.org/), and cats have been seen to consume other rodents of this size (Galão et al. 2025; Hernandez et al. 2018; Plimpton et al. 2021). *Microtus californicus* is a species of meadow vole native to California; though the species found in our study region is not of conservation concern, other regions contain endangered subspecies such as *M. californicus scirpensis* (Cudworth and Koprowski 2010) and more research may be needed on the relationship between cats and this species.

Domestic cats have been shown to exhibit significant individual variation in hunting behavior (Dickman and Newsome 2015; Moseby et al. 2015). Studies quantifying hunting behavior of indoor–outdoor cats using animal‐borne cameras have found that of cats that are studied, only a subset of cats ever exhibit hunting behavior, and an even smaller percentage consume the prey they captured (Hernandez et al. 2018; Loyd et al. 2013; Seymour et al. 2020). In fact, some studies even suggest that studying the impact of cats on wildlife on the population level can be misleading, and highlight the importance of focusing conservation efforts on the select number of individuals that hunt significant amounts of prey (Moseby et al. 2015; Thomas et al. 2012). Our data show that even among a small select number of indoor–outdoor cats surveyed for a short period of time (mean = 12.1 days), wild prey DNA was detected by diet metabarcoding. However, compared to studies that used this method to assess the diet of unowned cats (Plimpton et al. 2021; Swankhuisen et al. 2026), owned cats showed little consumption of wild prey, with only one sample containing wild prey DNA. A study conducted on a larger sample of cats for longer periods of time could illuminate the individual variation of hunting behavior of domestic cats, and our results show support for further assessing wild prey hunting and consumption by owned cats with a larger sample size.

It is worth noting that vole DNA did not remain in the gut of the cat for several consecutive samples over time. As reference, cats have a gastrointestinal transit time of 12–24 h and produce 1–2 scats per day, though there are interindividual differences (Telles et al. 2022); this suggests that prey DNA may only be retained for one digestion cycle upon consumption. However, it is possible that these time estimates reported by owners were not accurate, and given that there was only one observation of wild prey DNA in the scats, this finding should not be extrapolated beyond this study. More research is needed on the retention times of various amounts of prey in the cat digestive tract to better understand the resolution and efficacy of diet analyses of predators.

### 4.2 Provisioned Foods vs. Scats

Metabarcoding of cat food samples revealed chicken, pig, turkey, and a variety of fish. Consistent with our predictions, we detected chicken and pig DNA in all food samples, which was not surprising given that chicken, pig, and cattle are the most common animals to be found in pet foods (AAFCO Dog and Cat Food n.d.). While our study lacked the sample size and representation needed to systematically compare between types of pet foods, we found some differences in success of recovery of diet items post digestion across different types of foods that warrant mention. Interestingly, the two prescription foods (Hydrolyzed Protein and Kidney Care) showed the lowest rates of recovery of animal DNA in the scats; it is possible that due to the processing required for these formulae, such as protein hydrolyzation, the animal DNA found in these foods are already highly degraded and post‐digestion detection of these DNA was less likely. However, we caution readers from extrapolating this result given that we did not aim to systematically test the effect of different formulae on post‐digestion recovery rates. Regardless, despite being fed animal‐based cat foods, several of our cat scat samples showed no vertebrate DNA except cat; we suspect that this may either be due to the degradation of animal DNA in commercially available pet foods. Many studies assessing the diet of urban wildlife frequently encounter pet foods that are left outside as sources of anthropogenic food (Bateman and Fleming 2012; Caspi et al. 2025); it is therefore important to understand how commercially available pet foods are digested by animals, and if or how well prey DNA from pet foods are retained in scat samples. Studies involving feeding trials of different types of pet foods would enable a more systematic test of the detectability of these animal DNA pre‐ and post‐digestion.

### 4.3 Pooling Scats

Overall, we found that extracting DNA from individual samples led to a larger number of diet items recovered from scats than when these samples were pooled prior to extraction (Table 3). However, for some cats (cats B and E), there were no differences between these two protocols. Importantly, however, different diet items seemed to be differentially affected by pooling: common, anthropogenic food items such as chicken showed up regardless of pooling, but the only instance of a wild prey species in our study showed up only from the individually extracted sample. Thus, when research aims emphasize the detection of small, rarely consumed prey items, we suggest avoiding pooling multiple samples together prior to DNA extraction due to its potential to reduce the likelihood of detecting these species.

### 4.4 Limitations

As with most molecular analyses of diet, we recognize the limitations associated with this methodology, such as primer biases in amplification of certain taxa (Liu et al. 2023; Nichols et al. 2018); in particular, the 12S primers we used here perform very poorly on herptiles (Caspi et al. 2025). Given that cats have been seen hunting lizards by previous studies as well as in our study, we suspect that the lack of lizard DNA in our scat samples may be due to biased amplification of taxa due to our primers, rather than a true absence of lizard DNA and suggest future studies to incorporate morphological as well as molecular methods when assessing cat consumption of herptiles (Galão et al. 2025). Additionally, we found that pooled samples may result in reduced potential of detecting prey DNA, and suggest future studies to prioritize more biological replicates over technical replicates, similar to other studies (Mata et al. 2019). Finally, our study had a small sample size. Given that we recruited participants of this study from a previous study (Chung et al. 2024), our participants were not recruited specifically for this study and therefore may not have been incentivized to participate through the duration of the study. The parts of our data obtained directly from the owners, such as the date/time of scat collection, location of scat, its freshness, and whether/how the cat interacted with wild prey may have suffered from lack of reliability and various biases, such as social desirability bias affecting the owners' likelihood to report findings that they deem undesirable or inconsistent with our research objectives, and recall bias, affecting the owner's ability to recall older information (Bell et al. 2019; Larson 2019).

## 5 Conclusion

The majority of the scats in our study contained only anthropogenic sources of vertebrate DNA, but eight scats contained no vertebrate DNA at all other than cat DNA despite being fed animal‐based cat foods, suggesting that commercially available pet foods may contain mainly highly degraded DNA that is not detectable in scat post‐digestion using this methodology. Of 32 samples, only one scat showed wild prey DNA, which was not detected by the owner‐reported observations of hunting behavior and, in the cases where owners reported their cat handling wild, intact prey we detected no prey DNA in their scat, suggesting that consumption of significant prey parts is needed for detection in scat analysis. Lastly, pooling was not successful in detecting the one wild prey consumption event in our study, warranting caution in use of this method. Overall, consistent with other studies on free‐ranging cats (Plimpton et al. 2021), our study provides support for use of DNA metabarcoding as a method to of hunting behavior by cats when consumption of prey has occurred; however, we suggest that it may be best utilized in conjunction with other methods designed to capture instances of hunting without consumption, such as owner reports in questionnaires, providing support for targeting owned cats as well as unowned free‐roaming cats in future research on cat impacts on wildlife.

## Author Contributions

**Hee Jin Chung:** conceptualization (lead), data curation (lead), formal analysis (lead), investigation (lead), methodology (lead), project administration (equal), writing – original draft (lead), writing – review and editing (equal). **Stevi L. Vanderzwan:** investigation (supporting), methodology (supporting), project administration (equal), supervision (supporting). **Benjamin N. Sacks:** conceptualization (supporting), data curation (supporting), formal analysis (supporting), supervision (lead), writing – review and editing (equal).

## Funding

This work was supported by the Animal Welfare Institute's Christine Stevens Award.

## Conflicts of Interest

The authors declare no conflicts of interest.

## Supporting information


**Table S1:** 12S Plate maps for amplicaon PCR showing the samples, replicates, and negative controls that were included in the reaction. Each cell indicates a well on the PCR plate. Samples starting with “B” are extraction blanks.
**Table S2:** 16S Plate maps for amplicaon PCR showing the samples, replicates, and negative controls that were included in the reaction. Each cell indicates a well on the PCR plate. Samples starting with “B” are extraction blanks.
**Table S3:** Detailed data on the subject cats, collection times and locations, and amplification success for each scat and food samples collected for the study (*N* = 42 samples total).
**Table S4:** Total duration of the collection period for each cat surveyed (*N* = 8).
**Table S5:** Invertebrate diet items (16S) found in the sampled pet foods, individual scats, and pooled scat samples.
**Table S6:** Owner reported observations of hunting vs. Prey DNA recovered from scat using DNA Metabarcoding.
**Table S7:** Detailed nutritional information of cat food samples used in our study (*N* = 4).
**Table S8:** Number of raw sequence reads for each diet item found in cat foods vs. individual scat samples produced by cats that consumed that food type.
**Table S9:** Number of raw sequence reads for each diet item found in individually extracted and pooled scat samples.

## Data Availability

The data that support the findings are available in the Appendix of the article as Supplorting Information.
